# The effect of methamphetamine abuse on dental caries and periodontal diseases in an Eastern China city

**DOI:** 10.1186/s12903-017-0463-5

**Published:** 2018-01-10

**Authors:** Tao Ye, Dongliang Sun, Guangying Dong, Guangjie Xu, Ligang Wang, Jinjin Du, Pengcheng Ren, Shibin Yu

**Affiliations:** 1 State Key Laboratory of Military Stomatology, National Clinical Research Center for Oral Diseases, Shanxi Key Laboratory of Oral Diseases, School of Stomatology, the Fourth Military Medical University, Xi’an, Shaanxi 710032 People’s Republic of China; 2 Department of Stomatology, Chinese PLA 413 Hospital, Zhoushan, Zhejiang 316000 People’s Republic of China; 3 Tangdu Hospital, the Fourth Military Medical University, Xi’an, Shaanxi 710032 People’s Republic of China

**Keywords:** Illicit drugs, Methamphetamine, Oral health, Dental caries, Periodontal diseases

## Abstract

**Background:**

Dental diseases are among the most frequently reported health problems in drug abusers. However, few studies have been conducted on oral health of methamphetamine (meth) abusers in China. The aim of the present study was to investigate the caries and periodontal health profile of former meth abusers in Eastern China.

**Methods:**

A cross-sectional study was conducted on 162 former meth abusers in the male Zhoushan Compulsory Detoxification Center. A standardized questionnaire, which collected information about age, drug-use duration / pattern, oral hygiene habit and systemic diseases, was administered. Then, a dental examination was performed to investigate the severity of dental caries and periodontal diseases. In evaluating dental caries, the prevalence of dental caries, the scores of decayed teeth (DT), missing teeth (MT), filled teeth (FT), and decayed, missing, filled teeth (DMFT) were recorded. In evaluating periodontal diseases, community periodontal index (CPI), and the prevalence of gingival bleeding, dental calculus, periodontal pocket and loose teeth, were recorded. Additionally, the non-parametric test was adopted to analyze the potential risk factors via SPSS.

**Results:**

All the participants abused meth by inhalation. The mean scores of DT, MT, FT and DMFT in the former meth users were 2.72 ± 2.78, 3.07 ± 3.94, 0.33 ± 1.03 and 6.13 ± 5.20 respectively. The prevalence of gingival bleeding, dental calculus, periodontal pocket and loose teeth was 97.53%, 95.68%, 51.23% and 9.26% respectively. The DT, DMFT and CPI scores in those who had abused meth for longer than 4 years were significantly higher than those who abused for less than 4 years (*P* = 0.039, 0.045, *P <* 0.001, respectively). The DT score in those who brushed their teeth more than twice a day were significantly lower than those who brushed less (*P* = 0.018).

**Conclusions:**

The status of caries and periodontal diseases among former male meth users in Eastern China was poor. Prolonged drug abuse and lower frequency of tooth brushing may be the risk factors of their poor status of caries and periodontal diseases.

**Electronic supplementary material:**

The online version of this article (10.1186/s12903-017-0463-5) contains supplementary material, which is available to authorized users.

## Background

As a global problem, drug abuse is a serious public health concern [1]. Drug abuse increases social economic burden, crime rate, unemployment rate, and it incurs other social costs such as an increased threat to public safety [2, 3]. Methamphetamine (meth) abuse is a serious problem in the United States, Asia, Mexico, South America, Middle East and Australia [4]. In 2000, the World Health Organization reported that more than 35 million people worldwide use meth [5]. In 2005, the National Findings Report by the USA National Survey on Drug Use and Health stated that nearly 10.4 million people ages 12 years and older (4.3% of respondents) had used meth at least once in their lifetime [4]. Over the past two decades, drug abuse has also become an important social and public health issues in China [6], which has the largest population and imbalance of economic development around the country. In China, the number of drug users registered officially has increased 30-fold from 70,000 to 2,098,000 between 1990 and 2012 [7]. It was estimated that there were more than 14 million drug abusers in China at the end of 2014 [7], although lack of the detailed data about meth abuse. Whatever, there is an urgent need for a better health care for drug abusers in China.

Long-term drug abuse can induce abnormity of multiple organ systems and result in a variety of clinical manifestations, including disorders of the central nervous system, respiratory system, cardiovascular system, and stomatognathic system [8–13]. It can even result in death if not treated immediately [14, 15]. In the past several decades, intravenous drug use has been predominantly practised, which increased the risk of transmitting infectious diseases via blood contact, such as HIV, Hepatitis B or C [16]. Thus, above-mentioned systemic diseases are considerable public concern for drug users.

Oral health is an important part of general health. Poor oral health has been related to mortality, morbidity, coronary heart disease, poor nutrition, speech impediments, reduced employability, and poor self-image [17]. Oral health problems are among the most prevalent diseases related to illicit drug abuse, especially meth abuse [13, 17]. Dental patients abusing meth can present with poor oral hygiene, xerostomia, rampant caries, which was called “meth mouth” [18, 19]. Previous studies have shown that the oral health of meth abusers is usually worse than that of the general population [17, 18]. Currently, it is suggested that the higher prevalence of caries and periodontal diseases in meth abusers has a close relationship with hyposalivation, xerostomia, high carbohydrate diet, and poor oral hygiene [18–20], decreased body immunity and endocrine dysfunction [21]. However, in the literature, there is little information about the oral health status of Chinese meth abusers, especially in economically developed Eastern China area, which affect seriously the anti-drug policy-making and professional oral health management for drug abusers. The aim of the present study was to investigate the oral health status of meth users in a coastal city in Eastern China.

## Methods

### Participants

There was 199 male former drug users in Zhoushan Compulsory Detoxification Center in Zhejiang province, China, in October 2014. In all 199 former drug abusers, there were 162 (81.41%) meth only users, 7 (3.52%) heroin only users, 25 (12.56%) meth + heroin users, and 5 (2.51%) Meth + magu users. The 162 former meth only users were chosen as the sample population for this cross-sectional oral health survey. The study protocol was approved by the Ethics Committee of the School of Stomatology, Fourth Military Medical University. All of the subjects provided oral informed consent before the study.

### Data collection and questionnaire

Data collection for this pilot study involved 1) a standardized questionnaire (as shown in Additional file 1), administered by an experienced dentist (TY), who collected information about age, drug-use duration, drug-use pattern (intravenous injection, inhalation, drink or other ways), degree of education (elementary school, middle school, college or postgraduate), oral hygiene habit (brushing teeth more than twice / once or never per day, brushing teeth for more than 2 min / 1-2 min / less than 1 min per time, rinsing their mouths with tap water after every meal / every day / seldom / never), and systemic diseases (hepatitis, HIV, gastritis, heart disease, hypertension, or other diseases, the diseases determination will also refer to the medical examination report after entering the compulsory detoxification center), and 2) a comprehensive oral/dental examination performed by 4 dentists with independent dental clinic experience for more than 5 years (DLS, GJX, LGW and JJD). The questionnaire administrator and the oral health examiners were blinded to the other information of each participant. Oral health examinations using portable dental chair were conducted using the artificial light, dental probe, dental reflector and Community Periodontal Index (CPI) probe according to the WHO basic oral health survey methods (version 4). Before the examination, all the participants were asked to rinse mouth carefully with tap water. To avoid the effect of gingival bleeding on examination, caries was assessed before periodontal status. The DMFT index was used to evaluate the dental caries status based on the World Health Organization caries diagnostic criteria [22]. Periodontal condition was assessed using CPI [22]. Measurements were made using a CPI probe at 6 sites (mesio-buccal, mid-buccal, disto-buccal, disto-lingual, mid-lingual, and mesio-lingual) per tooth. The prevalence of gingival bleeding on probing, dental calculus, periodontal pocket and loose teeth was also calculated. To ensure the consistency of the examining results, 15 volunteers from dental students (grade III) in our school were chosen 1 week before the examination. After the training of the four dentists, all 15 volunteers were examined by every dentist, and the DMFT score and probing pocket depth were recorded and repeated within a 2-week interval by the 4 dentists. Intra-examiner (the consistency of examination results collected by the same dentist) and inter-examiner (the consistency of examination results collected by different dentists) agreements for the oral examination were good, as indicated by kappa statistics of more than 0.8.

### Statistical analysis

After a database of the results was set up, the SPSS 13.0 package (SPSS Inc., Chicago, IL, USA) was used to analyze and describe the data. Descriptive summaries were used to summarize the sociodemographics of the former meth users. To analyze the potential risk factors, all participants were divided into 2 groups according to age group (≤ or >33 years old, the median of age in all participants), the duration of drug abuse (< or ≥4 years, the median of the duration of drug abuse), tooth brushing times per day (< or ≥2 times), and tooth brushing time (≤1 min or 1-2 min per time). Because the present data did not follow normal distribution, a non-parametric test (Mann-Whitney U test) was adopted. *P*-values less than 0.05 were considered to be statistically significant.

## Results

### Demographic information

The mean age of the 162 former meth abusers was 33.67 ± 7.03 years, with the median 33.00 years old, ranging from 19 to 50 years old. The mean duration of illicit drug use was 5.20 ± 3.85 years, with the median 4.00 years, ranging from 1 to 20 years. Only 6.17% (7/162) of the former meth users had a college education, while others had at most a middle school education. All (100%) the participants abused meth by inhalation.

### Prevalence of systemic diseases

In all 162 participants, 36.41% (59/162) had systemic diseases. In all the diagnostic systemic diseases, gastritis (16.05%) and hepatitis (11.11%) were the most common diseases, followed by heart disease (6.79%) and hypertension (5.56%).

### Oral hygiene habit

Most of the participants (70.99%) brushed their teeth at most once a day, while only 29.01% brushed their teeth twice or more often a day. Forty participants (24.69%) brushed for less than 1 min, and the remaining participants (75.31%) brushed for 1–2 min. Only 38.27% of participants rinsed their mouths with tap water after every meal.

### Decayed, missing and filled teeth

As shown in Table 1, the mean DT, MT, FT and DMFT scores in the former meth abuse population were 2.72 ± 2.78, 3.07 ± 3.94, 0.33 ± 1.03 and 6.13 ± 5.20. The DT and DMFT scores in those who had abused drugs for longer than 4 years were significantly higher than in those with less than 4 years of abuse (Z = −2.089, *p* = 0.039; Z = −2.039, *p* = 0.045, respectively). Additionally, the DT scores in those who brushed their teeth more than twice per day, were significantly lower than those who brushed less (Z = −2.357, *p* = 0.018). Although the mean DMFT scores in those who brushed their teeth more than twice per day, were lower than those who brushed less, but no significant difference found (Z = −1.775, *p* = 0.076). However, the tooth brushing time had no significant association with the DT, MT, FT and DMFT scores.

**Table 1:** Association between Caries, periodontal status and age, drug abuse duration and tooth brushing habits of former meth users (*N* = 162)

		Sample	DT	MT	FT	DMFT	CPI
Age group	≤33 years old	84	2.88 ± 2.51	2.51 ± 3.23	0.29 ± 0.80	5.68 ± 4.61	2.40 ± 0.71
	>33 years old	78	2.53 ± 3.04	3.68 ± 4.53	0.39 ± 1.02	6.62 ± 5.78	2.87 ± 0.78**
Drug abuse duration	<4 years	76	2.17 ± 1.99	2.57 ± 2.64	0.25 ± 0.77	4.99 ± 3.47	2.39 ± 0.71
	≥4 years	86	3.20 ± 3.25*	3.52 ± 4.78	0.42 ± 1.21	7.14 ± 6.20*	2.84 ± 0.78**
Tooth brushing frequency	<2 times per day	115	3.03 ± 2.98	2.95 ± 3.19	0.38 ± 1.10	6.37 ± 4.79	2.64 ± 0.80
	≥2 times per day	47	1.94 ± 2.01*	3.38 ± 5.40	0.23 ± 0.81	5.55 ± 6.12	2.60 ± 0.74
Tooth brushing time	≤1 min per time	40	2.53 ± 2.56	3.33 ± 3.87	0.15 ± 0.58	6.00 ± 5.04	2.67 ± 0.80
	1–2 min per time	122	2.78 ± 2.85	2.99 ± 3.98	0.40 ± 1.13	6.17 ± 5.28	2.61 ± 0.78
Total	Mean score		2.72 ± 2.78	3.07 ± 3.94	0.33 ± 1.03	6.13 ± 5.20	2.63 ± 0.78
	Range		0–21	0–25	0–8	0–28	1-4
	Prevalence		79.01%	72.22%	14.20%	90.74%	–

*p < 0.05

**p < 0.01

### Periodontal diseases

The prevalence of gingival bleeding and dental calculus in all the participants was 97.53% (158/162) and 95.68% (155/162). Regarding the severity of dental calculus, grade I was determined in 29.63% (48/162) and grade II and grade III in 43.21% (70/162) and 22.84% (37/162), respectively. The prevalence of periodontal pocket and loose teeth was 51.23% (83/162) and 9.26% (15/162). The distributions of the CPI scores of 0, 1, 2, 3 and 4 were 0%, 3.09% (5/162), 46.30% (75/162), 35.19% (57/162), and 15.43% (25/162), respectively, with a mean CPI of 2.63 ± 0.78. As shown in Table 1, the CPI scores in those who had abused drugs for over 4 years were significantly higher than those with less than 4 years of abuse (Z = −3.789, *p* < 0.001). Additionally, the CPI scores in those who were older than 33 years of age were significantly higher than those who were younger than 33 years (Z = −3.850, *p* < 0.001).

## Discussion

Drug abusers belong to a special population with disordered emotions, behaviors and personalities [23]. It is suggested that the higher prevalence of oral diseases in meth abusers is closely related to xerostomia, high-carbohydrate diet, poor oral hygiene, and decreased body immunity and endocrine dysfunction [18–21]. The illicit drugs can stimulate α-adrenergic receptors within the salivary gland vasculature, causing vasoconstriction and reduction of salivary flow (hyposalivation), which greatly weakens protective properties such as the neutralization of plaque-induced acids and the remineralization of dental enamel [24]. Repetitive meth abuse can also lead to a decrease in salivary pH [25], which elevates the risk for dental erosion. In addition, Tipton DA found that meth can significantly increase bacterial lipopolysaccharide (LPS)-stimulated IL-1β levels secreted by monocyte/macrophages, which could contribute to periodontitis in meth abusers [26]. Furthermore, meth suppressed immune system activity in mice [21] and inhibited receptor-mediated phagocytosis, MHC class antigen II processing and antigen presentation [27]. Therefore, immune-weakening effects of meth might lead to increased inflammatory processes that will also involve periodontal tissues, particularly during chronic meth abuse.

Reported oral health surveys in the meth abusers show dental caries as the most commonly mentioned disease. In most of the oral health surveys, both the prevalence and severity of dental caries in meth abusers were higher than in the general population [18, 28, 29]. In a recent well-controlled survey in the United States (U.S), using a covariate-balancing propensity score strategy, meth users were twice as likely to have untreated caries and 4 times more likely to have caries than the control group of National Health and Nutrition Examination Survey (NHANES) participants [30]. Additionally, meth users were twice as likely to have 2 more decayed, missing, or filled teeth than the NHANES participants [30]. In the present study, the mean DT, MT and DMFT scores in the drug addicted population were all higher than in the corresponding epidemiological data of 35- to 44-year-old individuals in Eastern China in the third National Epidemiological Sampling Survey of Oral Health (NESSOH) [31], which is consistent with the results of previous reports [18, 28, 29]. In addition, the significantly higher DT and slightly lower FT scores in the former meth abusers indicated that meth abusers usually neglect their oral health care.

Periodontal disease is another commonly mentioned disease in the reported oral health surveys in the drug addicted population. In the present study, the prevalence of gingival bleeding, periodontal pockets and deep periodontal pocket in former meth abusers was higher than the corresponding reference data in the third NESSOH [31]. The Furthermore, a high mean CPI was found in the present study, which is similar to the previous CPI data in meth abusers [28].

It has been suggested that those with a longer drug use history had a higher risk of developing bad oral health [32–34]. In the present study, the DT, DMFT and CPI scores in those who had abused meth for longer than 4 years were significantly higher than those who had a shorter abuse history, which is consistent with previous reports. It indicates that shorter meth abuse is better for the status of caries and periodontal diseases.

The life-style of drug abusers is usually different from that of the general population. They usually ignore their oral health and have poor oral hygiene [18–20, 28, 35–37]. In the present study, the DT score in those who brushed their teeth more than twice a day, were significantly lower than those who brushed less frequently, which demonstrated that good tooth brushing habits play an important role in the maintenance of oral health of meth abusers. It coincides with a previous suggestion that the higher prevalence of caries in meth abusers is closely related with poor oral hygiene [18–20].

Inhalation and intravenous injection are the 2 main intake patterns of illicit drug abuse. When injecting drugs intravenously, the risk of transmitting infectious diseases (such as HIV, Hepatitis B or C) via blood contact is high, while inhalation will lead to significant strain on the lungs and the respiratory tract [16]. Generally speaking, inhalation is significantly less dangerous. In the present survey, all former meth abusers abused drug by inhalation, which is consistent with several recent surveys [17, 38, 39]. China has substantial differences in HBsAg prevalence across regions, ranging from 2% to 15%, with the national average of 7.18% [40]. In the present survey, although about 1/3 of the participants had some kind of systemic diseases, about tenth of the participants had hepatitis (including all kinds of hepatitis), and there was no participant with HIV infection. The inhalation intake pattern seems to be the main reason why the prevalence of hepatitis and HIV was not significantly higher than that of general population.

The present study has several limitations. First, due to the harsh China’s drug policy, we can only find drug abusers in Compulsory Detoxification Center through normal channels. So, we can’t control the sample size freely, and the present sample is a convenient sample. Second, all the participants were from a Compulsory Detoxification Center, which means that they were former drug abusers. Third, both the duration of their drug abuse in the past, and the duration of their drug detoxification and rehabilitation were different, which may affect the results. Forth, in the present survey, the age of the participants ranged from 19 to 50 years old. The reference data from 35 to 44 years age group in East China in the third NESSOH [31] were used as reference control. The age difference may have some effect on the present results.

## Conclusions

The status of caries and periodontal diseases among former male drug users in Eastern China was poor. Prolonged drug abuse and lower frequency of tooth brushing may be the risk factors of their poor status of caries and periodontal diseases. Abstaining from drugs as early as possible and good oral hygiene habit may contribute to good status of caries and periodontal diseases in meth abusers.

## Additional file

Additional file 1: Questionnaire-Questionnaire Related with Oral Health-the questionnaire used in the present survey. (DOCX 12 kb)

## Abbreviations

CPI: Community periodontal index
DMFT: Decayed, missing and filled teeth
DT: Decayed teeth
MT: Missing teeth
NESSOR: National Epidemiological Sampling Survey of Oral Health
NHANES: National Health and Nutrition Examination Survey
