# Supplementary material for: The effect of methamphetamine abuse on dental caries and periodontal diseases in an Eastern China city
Source: BMC Oral Health. 2018 Jan 10;18:8. doi: 10.1186/s12903-017-0463-5 (PMC5763656; doi:10.1186/s12903-017-0463-5)
Supplement: Additional file 1: — Questionnaire-Questionnaire Related with Oral Health-the questionnaire used in the present survey. (DOCX 12 kb) [file 12903_2017_463_MOESM1_ESM.docx]

**Questionnaire Related with Oral Health**

( To get the real information about your oral health, please read the followed questions and answer carefully!)

No. of Participants: Name: Age: years old

1. Degree of education: ①Elementary school ②Middle school

③College ④ Postgraduate

2. Duration of previous drug-abuse: years

3. Type of abused drugs:

4. Pattern drug-abuse: ①Intravenous injection ② Inhalation

③Drink ④ Other ways

5. How many times do you usually brush your teeth every day?

①≧3 times ② 2 times ③ once ④ less than once

6. How many minutes do you usually brush your teeth for per time?

①≧3 minutes ② 2-3 minutes ③1-2 minutes ④ less than 1 minute

7. How often do you rinse your mouth with tap water after meal?

① Rinse mouth after every meal ② Rinse mouth every day

③ Seldom rinse mouth ④ Never rinse mouth

8. Do you have systemic diseases?

① Hepatitis ②HIV ③ Gastritis ④ Heart disease

⑤ Hypertension ⑥Other diseases
